# Metal Artifact Reduction in Spectral X-ray CT Using Spectral Deep Learning

**DOI:** 10.3390/jimaging8030077

**Published:** 2022-03-17

**Authors:** Matteo Busi, Christian Kehl, Jeppe R. Frisvad, Ulrik L. Olsen

**Affiliations:** Department of Physics, Technical University of Denmark, 2800 Kongens Lyngby, Denmark; c.kehl@uu.nl (C.K.); jerf@dtu.dk (J.R.F.); ullu@dtu.dk (U.L.O.)

**Keywords:** spectral convolutional neural networks, spectral X-ray CT, metal artifact reduction, computed tomography, non-destructive evaluation, spectral deep learning

## Abstract

Spectral X-ray computed tomography (SCT) is an emerging method for non-destructive imaging of the inner structure of materials. Compared with the conventional X-ray CT, this technique provides spectral photon energy resolution in a finite number of energy channels, adding a new dimension to the reconstructed volumes and images. While this mitigates energy-dependent distortions such as beam hardening, metal artifacts due to photon starvation effects are still present, especially for low-energy channels where the attenuation coefficients are higher. We present a correction method for metal artifact reduction in SCT that is based on spectral deep learning. The correction efficiently reduces streaking artifacts in all the energy channels measured. We show that the additional information in the energy domain provides relevance for restoring the quality of low-energy reconstruction affected by metal artifacts. The correction method is parameter free and only takes around 15 ms per energy channel, satisfying near-real time requirement of industrial scanners.

## 1. Introduction

Spectral X-ray computed tomography (SCT) is an emerging technique for enhanced non-destructive investigation of inner features of objects. In SCT, the energy dependence of the linear attenuation coefficient (LAC) of materials is measured with the aid of detectors able to discriminate the energy of the incoming photons in a discrete number of energy channels. It has been demonstrated that using this technique yields enhancement of the contrast-to-noise ratio (CNR) [1,2,3,4], the characterization of materials [5,6] and the discrimination of threat objects [7,8]. However, this technique still suffers from degrading effects, such as photon starvation, caused by the presence of high attenuation materials (e.g., metals). This effect yields severe streaking artifacts in the reconstructions that typically originate from a metallic object and extend further, overlapping with the other objects or materials present in the volume. The metal artifacts not only degrade the graphic quality of the reconstructions, but also additionally challenge the quality of segmentation and thus, the classification and characterization of materials. For these reasons, metal artifact reduction (MAR) methods are key to the accurate and precise performance of a CT scanner both in medical [9,10] and non-destructive testing fields [11,12].

The goal of a MAR algorithm is to improve the quality of the reconstructions by removing the streaks while preserving the real features. This can be conducted in a pre-reconstruction approach by applying a correction algorithm in the sinogram domain. This is typically conducted by adaptive interpolation of the photon-starved detector pixels in the projections. Alternatively, MAR can be conducted in a post-reconstruction step, by directly attempting to remove the streaks and restore the LAC values of the materials. However, these traditional correction methods typically require a high amount of prior knowledge for iterative regularization, are tailored ad-hoc for certain applications, and fail to offer a generalizable solution. Convolutional neural network (CNN) architectures have shown a lot of success recently in fields such as computer vision and image classification and segmentation [13,14,15]. In the field of X-ray CT imaging, several studies using deep learning methods have shown promising results in MAR [16,17,18], scattering correction [19,20], ring artifact reduction [21], and reconstruction in low-dose regimes [22,23]. However, most of this work is restricted to conventional energy-integrating CT applications. When SCT is considered, these architectures and algorithms can be used for each energy channel individually, but this easily becomes inefficient and would neglect trends and signal coherence embedded in the spectral domain.

In order to include spectral domain information, we adopt a CNN architecture designed for biomedical image segmentation, namely U-Net [24]. This is an encoder-decoder network type. The encoder sub-network takes an image as input and recursively generates a higher dimensional vector of feature maps (with information on the features of the input image). The decoder sub-network takes the feature maps and inverts the steps to reconstruct an image in the same domain as the input. Metal artifacts are imaging artifacts that span the entire spectral domain, meaning that the spectral channels are semantically interconnected. Therefore, in order to recover structural information in lower energy spectra from higher energy spectra, we need to connect the channels, and thus need to perform the 3D convolution—a 2D, per-spectrum imaging filtering disassociates the spectra and thus does not yield the target effect. Çiçek et al. [25] presented a 3D U-Net architecture for volumetric segmentation, which replaces all the 2D operations with their 3D counterparts. In this work, we use a similar architecture for 3D data, but we use the third dimension for the energy domain. To the best of our knowledge, this has not been done before.

The importance of the information in the spectral domain has been confirmed in related work. A CNN-based SCT method that includes information in the spectral domain is that of Wu et al. [26]. This method is designed to obtain an improved SCT reconstruction if the signal is Poisson noisy, but MAR is not addressed. A few CNN-based SCT methods do address MAR [27,28]. They use a network with a U-Net architecture to take a dual energy signal and convert it (in different ways) into a virtual monochromatic image with significantly reduced metal artifacts. These methods were only tested in simulations and the spectral information was traded for MAR. We propose a method that retains the spectral information while also achieving MAR. The method is conceived and can be applied to general SCT applications; however, in this manuscript we fine-tune the training and evaluate experimental data in particular for use in luggage scanners.

## 2. Methods and Techniques

**Figure 1:**
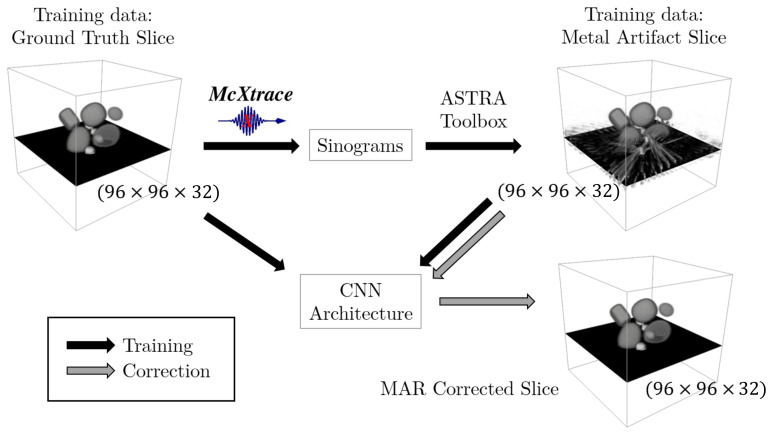
A sketch of the MAR correction using spectral CNN. The 3D volume of the ground truth phantom of the materials is given as input to the McXtrace software package to obtain physically modeled spectral sinograms via X-ray tracing simulations. These are reconstructed into metal artifact affected volume reconstruction, using the ASTRA [29] toolbox. For each vertical slice of the object’s 3D volume, a pair of ground truth and reconstructed spectral volumes (where the third dimension is now the energy channel) is given as input training data in the CNN architecture (black arrow). Once trained, the CNN architecture corrects directly the metal-artifact affected spectral slices (gray arrow). The metal objects are visible as the lighter gray items.

The overarching goal of the method presented in this manuscript is to remove the metal streaking artifacts from energy-resolved reconstructions measured using SCT. In SCT, the level of photon starvation depends on the LAC properties of the materials. It is thus an energy dependent phenomenon and there may be samples for which the artifacts appear in the low-energy channels but not in the high-energy ones. This is of great value as the information regarding geometrical features, shapes and material physical properties can be extracted from the artifact-free high-energy domain and used to restore the reconstruction in the artifact-affected low-energy domain. In our approach, the MAR is performed in a post-reconstruction step with the aid of a spectral CNN architecture. The CNN is trained using a data pair consisting of the metal artifact-affected and ground truth (artifact-free) reconstructions. Once trained, the CNN can directly correct metal artifacts from reconstructions. Figure 1 summarizes our approach in a block diagram.

One of the key factors to the success of the CNN corrections is the quality of the training data, i.e., how well the samples generalize the problem to be solved. With our architecture, an image set for training is a pair of distorted and ground truth spectral images, which, in a real acquisition, correspond to the measured and desired corrected images. Depending on the difficulty (in terms of generalization) of the problems that we aim at solving using a CNN, the quantity and variety of the training data must be adjusted. In the literature, as a rule of thumb, at least a few thousand sets of training data are required to model realistic, non-trivial imaging problems. Such a large number is generally achieved by relatively simple data augmentation steps, in which elementary operations such as Gaussian noise filtering and affine transformations are applied to the input data. The training data can either be generated starting from real experimental data or simulation data. Alternatively, efficient solutions have been found by a first step training with synthesized simulation data, followed by a second training step with experimentally measured data. For most applications, experimental training data are hard and slow to generate in sufficient amounts. For metal artifact corrections, for example, the ground truth would be the results of iterative correction algorithms or manual correction of the measured data.

In the following, we present a method for generating training data based on physical models of photon-matter interactions (Section 2.1) and then our spectral CNN architecture (Section 2.2).

### 2.1. Training Data

**Figure 2:**
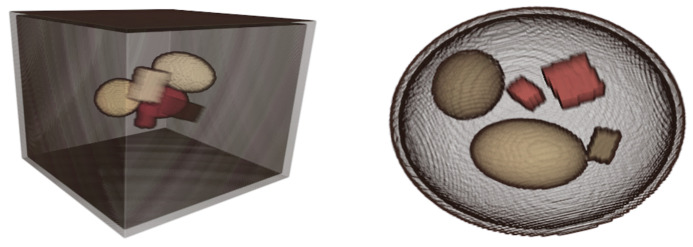
Two 3D representations of the randomly generated phantoms. A cuboid (**left**) and an ellipsoid (**right**) envelope, containing objects with randomly chosen shape, size, center coordinates and material composition.

In this work, the ground truth training data are generated with the aid of a software tool for CT phantoms presented by Kazantsev et al. [30]. Using this tool, we generated artificial phantoms composed of different materials using randomly-parametrized elementary shape geometries, such as ellipsoids, parallelepipeds and cubes, which are represented as implicit surfaces. The materials are randomly chosen from a list of materials that can be commonly found in luggage and include at least two metals. The LACs of the materials were obtained with a combination of in-house spectral LAC measurements of materials with known density, and entries from the XCOM database [31]. In addition, each phantom includes multiple metallic objects of either copper, gold or tungsten, to enforce the appearance of metal artifacts in the reconstructions. Figure 2 shows a few realizations of random phantoms.

The metal artifact-affected reconstruction are produced via modeling the forward projection with the sample component for SCT simulations presented by Busi et al. [32]. The toolkit is powered by McXtrace [33], a software package for Monte Carlo (MC) X-ray tracing simulations and experiments. The ground truth phantoms are given as input and used to simulate the energy-resolved projections. The scattering noise is ignored and incorporated in the photon cross section attenuation to save computational time in the simulations. Each energy-resolved sinogram is reconstructed channel-by-channel using the CUDA version of the SIRT iterative reconstruction algorithm in the ASTRA toolbox [29]. The reconstructions match with their respective ground truth but they have streaking metal artifacts, due to the inclusion of the photon statistics in the simulation. The statistical noise leads to fluctuations from a noise-free value, which are stronger for metals than for lower Z (atomic number) elements. Thus, we get streaking in the reconstructions because we do not simulate the 3D volume but individual projections.

Once the full set of the training data designed for the CNN learning is generated, the dataset pairs are split and grouped into training data, validation data and test data. The training data consists of the samples that are used by the network to fit the parameters at each epoch. The validation data are excluded from the training of the network and used after the fitting at each epoch to tune hidden-layer parameters, avoid overfitting and evaluate the convergence of the training. Similarly, the test data consists of samples that are excluded from the training and are only used at the end of the overall training of the network to analyze the performance of the trained CNN architecture in solving the assigned problem. Experts in the field typically recommend to randomly divide the dataset into 64% training data, 16% validation data and 20% test data [34]. The main advantage of this method is that the ground truth is intrinsically available as it is part of the input requirements of the simulations. In addition, the software package can be set such that it uninterruptedly generates new training data with none, or minimal operator intervention required. While it is generally recommended to use exclusively real training data, as it is the best representation of the problem to be solved, it was not possible to obtain experimental ground truth images. As such, the networks were fully trained with simulation data and the corrections were tested on both real and synthetic data.

### 2.2. Spectral CNN Architecture

As this work focuses on spectral X-ray CT, the U-Net architecture present in literature required an adaptation that enables the extraction of the additional information in the energy domain as well as to support three-dimensional data. This is a technical and conceptual challenge because the spectral images are represented as large 3D matrices (e.g., 256×256 pixels, 128 energy channels, 32-bit floating-point pixel values), each of which connected to its chain of weight maps for each network filter (i.e., the sub-unit of a layer), which have to be kept in the memory of the graphics card at the same time. The architecture adopted in this work for SCT contains 3D convolutional layers that can extract information from the spectral domain.

**Figure 3:**
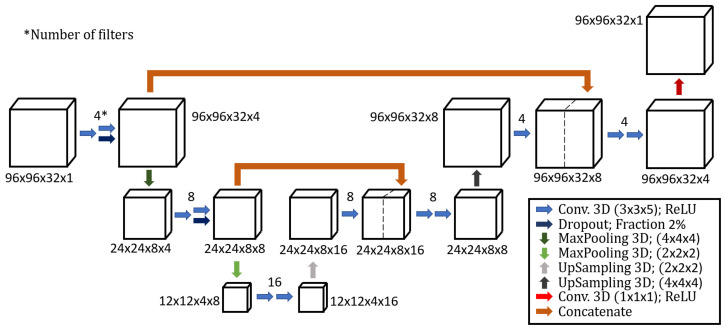
Sketch of the 3D U-Net architecture, with all the building blocks and filter types. The legend inset in the lower right hand side corner shows the color code for the activation functions.

Figure 3 illustrates the 3D U-Net architecture built using Keras [35], a Python deep learning library.

The network contains several layers of parameters that are recursively updated by loss function optimization. The update of the parameters β can be expressed as:

(1) $$\beta \prime =\underset{\beta }{\arg\min}L\left(\beta \right)+\lambda \Gamma \left(\beta \right),$$

where λΓ(β) is the regularization (or penalty) term and L is the loss function:

(2) $$L\left(\beta \right)=\frac{1}{n}\sum_{i=1}^{n}L\left(y^{i},f\left(x^{i},\beta \right)\right).$$

Therein, *f* is the activation function (or filter), which expresses how the input values $x^{i}$ are related to the network predicted values ${\tilde{y}}^{i}$. *L* represents the difference between the network’s prediction and the target $y^{i}$, and can be expressed in many ways (e.g., mean squared error, mean squared logarithmic error, mean absolute error). The filter types used by the CNN architecture appearing in the figure are listed and described below:

- Conv. 3D: These are layers of the network consisting of a defined number of 3D convolutional filters with defined size. These filters are the main units (or neurons) of the network as they contain all the coefficients being tuned during the training process. Note that the number of filters at each step defines the fourth dimension of the incoming dataset in Figure 3. The Rectified Linear Units (ReLU) apply the function f(x)=max(0,x) to all the values in the input volume, which replaces all the negative activation with zeros. ReLU is applied after each convolutional layer to introduce non-linearity.
- Dropout: During training, some training entities (i.e., pixels) are randomly chosen and ignored or, in other terms, dropped. This step is introduced to avoid overfitting, as suggested by Srivastava et al. [36].
- MaxPooling: Max pooling refers to the step of applying a maximum filter to (usually) non-overlapping sub-regions of the initial representation, and it is necessary to reduce the dimension of the data while preserving the features. For example, a 3D MaxPooling with size (2×2×2) halves the size data in each of the three dimensions.
- UpSampling: Works as opposite of the MaxPooling operation. By default, this is conducted by padding the matrix cells with zero values. Alternatively, the upsampling can be carried out with interpolation.
- Concatenate: This step concatenates datasets along a chosen dimension. For example, the concatenation in the first layer of the CNN architecture concatenates the data preceding the MaxPooling and the data connected to the 4 filters convolutional layers, both with a size of (96×96×32×4), into a single data with size (96×96×32×8).

Adam [37] was employed as the optimization algorithm, and the initial learning rate was set to $1.0\times 10^{-4}$ and the decay was fixed at $3.0\times 10^{-7}$. The learning rate determines how quickly the network parameters are updated at each step of the gradient descent whereas the decay represents the diminishing of the learning rate after each update.

In this work, the initial spectral image was with size (96×96×32) although the network can be adjusted to fit different image sizes. The image size chosen for this work was relatively small to allow for a fast check of results with modifications of the network and the training data. We avoid patch-based training, where smaller patches of the initial images are used as input, because the image distortions and artifacts (which our method corrects) are found in correlations across the whole image and thus represents non-local pixel relations. Additionally, batch normalization is not performed because the network operates with training data that have a globally variable value range. Instead, the image values are converted into either attenuation or absorption via Lambert-Beer’s law. The batch size of the network itself was set to 32 to satisfy constraints of the available hardware and the number of parallel worker processes employed for data loading and augmentation.

The drawback of a 3D network like ours, and the limiting factor for applications to physically measured data with large image sizes, is the exponential computation requirement. Existing hardware limitations govern the maximal depth of a 3D U-Net implementation, which prohibits the learning of higher-level logic and global data relations across the image. The hardware limitations can be overcome in trivial scenarios by increased pooling sizes, leading to a more aggressive downsampling of the input image data and a reduction in the number of learning filters. This approach has limitations in practical implementations when a given minimum observation size for imaged features is required and when a minimum number of features is to be extracted for the image correction. We observed a significant loss of detail in the image correction due to hardware resources restricting the 3D network to be relatively shallow (i.e., very few down/up sampling blocks) and operating at excessively coarse resolution. A possible solution to this would be the integration and subsequent combination of alternative architectures able to preserve resolution and detail with respect to the spatial patterns as well as the spectral signal response of the data.

### 2.3. Issues Faced in MAR Development

The main issue faced when including highly attenuating materials in the training data is the logarithmic nature of the LAC of materials. Since the metals have a value range that is significantly superior to organic compounds, there is a biased training of the CNN architecture. That is because the training relies on the tuning of convolutional filters by minimizing a loss function, which is the difference between the two input images. Due to photon statistic noise, the difference between the simulated and ground truth image is significantly higher for the metal when compared with organic compounds. Consequently, low-attenuation materials vanish from the images, as they are seen by the architecture as a noisy void, hence replaced with zero values. On the other hand, in most cases the overarching goal of the correction is not a mere streaking reduction but a restoration of the image features of the low attenuating materials to efficiently perform further analysis routines. The solution proposed and used in this work is a high attenuation thresholding, which consists in substituting LAC values greater than the LAC of silicon with the corresponding values of silicon. This step is carried out directly after the tomographic reconstruction and is justified by the fact that in most SCT application the metals are not the objective of the data analysis but an intrusive material that leads to artifacts. The CNN architectures are optimized to work with data normalized in the value range $\left[0,1\right]$. Thus, the data undergoes an additional normalization step in which each individual energy-dependent projection is remapped to the value range $\left[\min,\max\right]\to \left[0,1\right]$, and we observed that this greatly improved the performance of the correction and the convergence speed during training.

A second concern in the data generation process is that the geometric shapes have great impact on the performance of the correction itself. Depending on the application of the CT itself, this imposes different conditions on the type of training data. For security screening scanners, ideally the CNN should have enough training data to learn all the possible geometries. Of course, this is limited by the storage and computational time to generate enough training data. In this work, the sample variation is restricted to relatively simple geometries and the few entries for the number of projections preceding the reconstruction. In an application focusing on luggage screening, objects could be scanned separately (including material characterization and segmentation) to provide the simulation inputs with realistic shapes for objects made of different materials.

Lastly, the aim of our MAR method is not only to restore the image quality by removing streaks and enhancing contrast between different material (as in most existing work). Our goal is also quantitative material characterization from physical properties. The preservation and restoration of the effective LAC values is therefore important too. The correctness of the physical models for generation of the training data thus becomes crucial as incorrect models may introduce offsets in the material LACs after the MAR correction has been applied.

## 3. Experiments

In this section, we present our MAR experiments with simulation data (Section 3.1) and with a real measurement of a suitcase composed of many metal parts (Section 3.2).

### 3.1. Simulation Test Data

For the benchmarking of the spectral MAR correction using CNN, the training data was generated using McXtrace with the following procedure. The number of individual unique phantoms was set to 10, and a SCT scan of each was conducted simulating randomly 120, 90, 72, 60 or 52 projections with a cone-beam geometry collimation of the source and a 256×256 flat-panel 2D spectral detector with 32 energy channels and a detection area of 204.8mm×204.8mm (the choice is to match our available real detector specifics). The energy channels are evenly distributed between 20 and 160 keV. The source-to-sample and detector distances were set respectively to 816.6mm and 1141.9mm. This led to a relatively high sample-to-detector distance, in order to minimize the scattering noise from the sample for the real experiment planned as a successive step. Each energy-resolved sinogram was reconstructed into a 3D volume using the ASTRA toolbox [29] and each training dataset was extracted as a pair of slices of input ground truth and reconstructed 3D volumes of the sample (see Figure 1). This resulted in a total of 925 training datasets split into training, validation and test as described above. Note that not every slice has visible metal artifacts due to the random composition of the phantom. Each slice pair was resized into the dimension (96×96×32), by a spatial downsampling using bicubic interpolation, to fit the CNN architecture and followed the normalization procedure described above.

**Figure 4:**
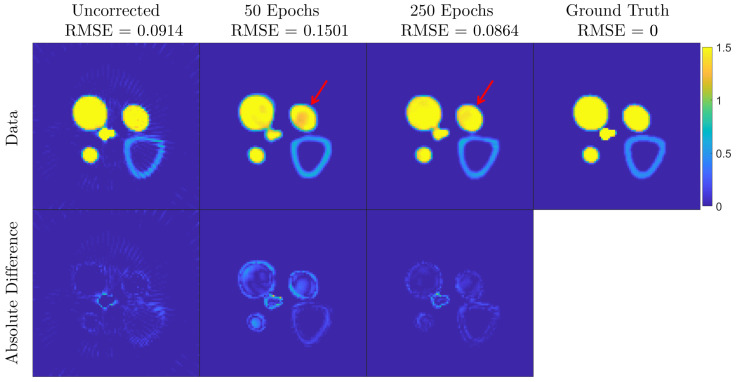
MAR correction analysis of a simulation test training data for different amounts of training epochs. The top row features the images for uncorrected data and corrected data, training the network for 50 and 250 epochs, and the ground truth as comparison reference. The bottom row features the absolute difference between the top adjacent images and the ground truth. The red arrow indicates the cupping artifact induced by the CNN correction when the network is trained for 50 epochs. This effect disappears when the network is trained for 250 epochs.

The training was carried out for 50 and 250 epochs, for a total time of approximately 2 and 8 h respectively, to analyze the training performance against the number of training epochs. Once trained, each metal artifact-affected slice is corrected in approximately 15ms, satisfying the near real-time requirement of industrial applications. Figure 4 features a comparison of two images, for the 29.4 keV energy channel, after training for 50 and 250 epochs. Observing the lower-bound 50 iterations, the results demonstrate that the CNN struggles to define whether an object should be full or hollow as modeled in the input data. This results in a cupping effect that is seen at 50 training epochs but disappears at 250 training epochs.

The performance of the correction is quantified in terms of the following metrics for image quality:

- Root Mean Squared Error (RMSE):
  (3) $$\mathrm{RMSE}=\sqrt{\frac{1}{N_{\mathrm{pix}}}\sum_{N_{\mathrm{pix}}}{\left(\mu^{\mathrm{gt}}-\mu^{\mathrm{CNN}}\right)}^{2}},$$
  where $N_{\mathrm{pix}}$ is the number of pixels in the ground truth ($\mu^{\mathrm{gt}}$) and CNN corrected ($\mu^{\mathrm{CNN}}$) energy resolved reconstructions.
- Normalized Root Mean Squared Error (NRMSE):
  (4) $$\mathrm{NRMSE}=\frac{\sqrt{\frac{1}{N_{\mathrm{pix}}}\sum_{N_{\mathrm{pix}}}{\left(\mu^{\mathrm{gt}}-\mu^{\mathrm{CNN}}\right)}^{2}}}{\langle \mu^{\mathrm{gt}}\rangle },$$
  which is RMSE divided by the mean of the ground truth pixel values.
- Mean Structural Similarity (MSSIM). Structural Similarity (SSIM) is a method for measuring the similarity between two images presented by Wang et al. [38]. This method returns an image with the same size as the input images, with values ranging from −1 to 1 taking maximum value when the two images are identical. In the MSSIM, a single index value is calculated as the mean value over the SSIM image.

These are calculated for each uncorrected and corrected (for both 50 and 250 training epochs) test data and reported in Table 1. The values reported in the table are calculated as the mean of all test data.

The MSSIM increases significantly for both the 50 and 250 epochs trained CNN corrections, meaning that the streaks are successfully corrected in most cases. The worse NRMSE found for 50 epochs can be caused by either lack of training data diversity or most probably due to the fact that the CNN training has not converged yet. For the rest of the manuscript, the CNN was trained for 250 epochs for a total time of approximately 8 h using a desktop PC equipped with NVIDIA Titan X (Pascal) and GeForce GTX 1080 GPUs, 256 GB RAM and an Intel(R) Xeon(R) E5-2637 v3 CPU.

#### Results and Discussion

**Figure 5:**
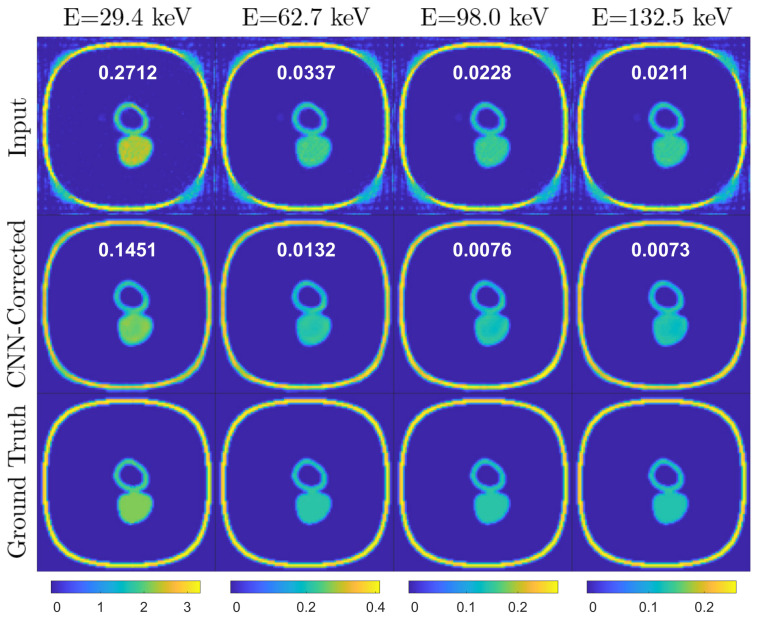
Simulation test data. Example 1: slice of the reconstructed volume at different energy channels when metal artifact affected (first row), MAR corrected (second row) and ground truth (third row). Each column corresponds to a fixed energy channel and they were plotted using the same color map. The color bar represents the LAC values. The inset number in the graphs reflects the RMSE value.

**Figure 6:**
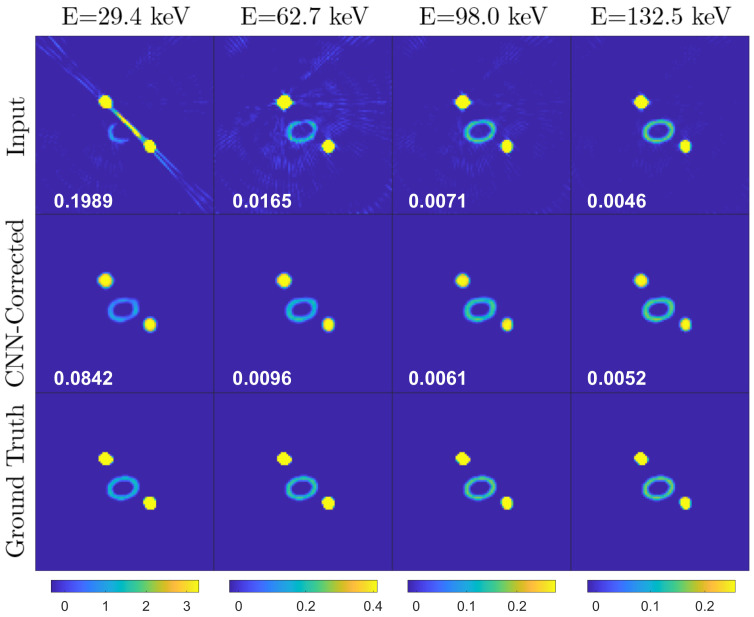
Simulation test data. Example 2: the object feature obscured by the metal-provoked streak artifact is restored by the CNN correction. (Layout as in Figure 5).

**Figure 7:**
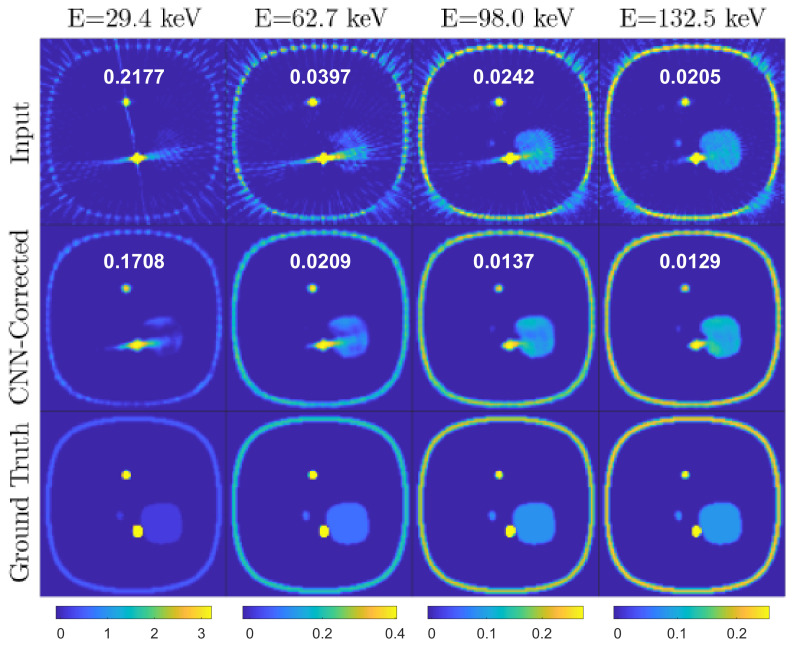
Simulation test data. Example 3: the object features are only partially corrected by the CNN architecture due to severe artifacts. (Layout as in Figure 5).

Figure 5, Figure 6 and Figure 7 represent a few examples of the MAR correction result.

In these figures, the top image is consisting of metal artifact-affected (Input), corrected (CNN Correction) and reference input (Ground Truth) image slices at four of the thirtytwo energy channels, corresponding to 29.4keV, 62.7keV, 98.0keV and 132.5keV. Note that the color map in the images is identical for each energy channel (column).

The first example (Figure 5) is a slice where moderate metal artifacts are visible in the first energy channel input images and in the vertical line profiles as an increase of the LAC with respect to the ground truth. Note that the correction efficiently restores the LAC values and smoothens the noise oscillations caused by either the presence of a metal in neighboring slices or the limited number of projections. The drawback is an introduction of moderate cupping, which could be solved by training the network for more epochs as discussed above. The second example (Figure 6) displays in the first energy channel a strong streaking metal artifact, which overlaps with a neighboring object provoking a partial disappearance of its geometrical features. The artifacts decrease in intensity as the energy increases, to a point where the shapes become clearly visible. This case demonstrates the advantage of spectral X-ray CT over conventional techniques as the CNN architecture utilizes the information on the material LAC and shape in the higher energy channel to restore the image quality in the metal artifact affected low-energy channels. However, in cases like the third example (Figure 7), where the LAC values of the compounds affected by the streaks are lower (compare the LAC in the vertical axes in Figure 6 and Figure 7), the streaks extend to higher energy channels, challenging the performance of the MAR correction. Nevertheless, the correction still succeeds in removing the streaks, leading to an enhanced image quality.

### 3.2. Experimental Test Data

The same CNN architecture that was trained with MC simulation data was then tested with real metal artifact-affected datasets. A suitcase composed of several metal parts was filled with objects commonly found in luggage and scanned with the spectral X-ray CT technique. The geometry and source settings were set to resemble the one in the simulations to generate training data and 75 projections were taken. As opposed to the simulations, the source was collimated into a fan-beam geometry to have a reduced amount of scattering noise while still having streaking artifacts due to metals. Each vertical slice of the object was measured by vertically translating the sample stage. The difference between the two reconstruction geometries is that vertical slices from the cone-beam reconstruction may have artifacts that are caused by metals belonging to adjacent slices, whereas this is not found for fan-beam geometry. The measured data processing routine followed the SCT procedure described by Busi et al. [6], rebinning the energy channels into 32 to match the simulation data. The adopted reconstruction algorithm was an iterative ART with TV regularization, presented by Sidky et al. [39], which was proven particularly efficient in case of few-projection CT scans. Each reconstruction slice was corrected using the CNN trained for 250 epochs and merged to reform the 3D volume stack.

#### Results and Discussion

**Figure 8:**
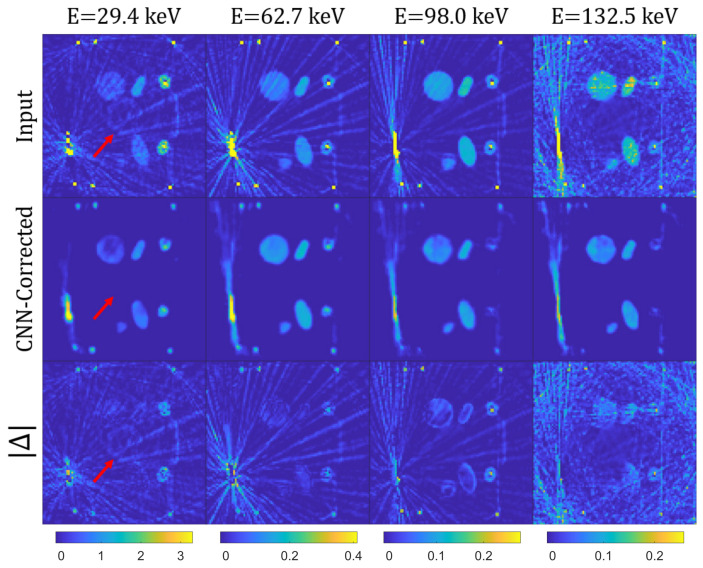
Slice of the suitcase for the input (first row) and CNN-corrected (second row) data at different energies and the absolute difference between the two (third row). Note that each column, corresponding to a fixed energy channel, has identical color map. The red arrow indicates a hollow object look-like artifact, which does not appear in the higher energy channel and is efficiently removed by the correction.

**Figure 9:**
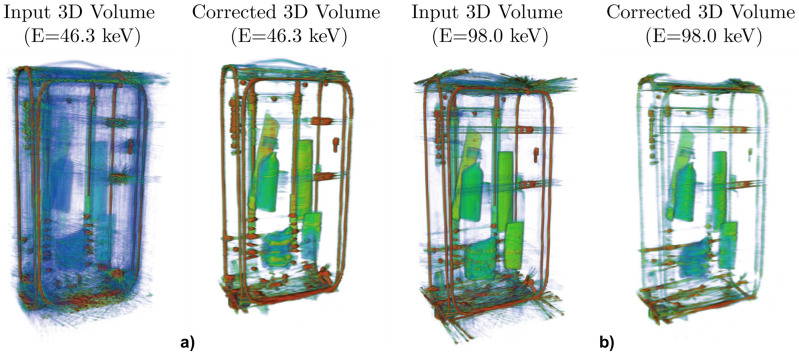
Input and MAR corrected 3D volume reconstruction of the suitcase at 46.3keV in (**a**) and at 98.0keV in (**b**). The CNN correction clearly reduces most of the artifacts increasing dramatically the quality of the reconstruction.

Figure 8 and Figure 9 display slices and 3D volumes of the uncorrected and corrected suitcase, obtained by stacking each vertical slice. Note that the MAR correction effectively cleans the reconstructions from most of the streaking metal artifacts, except for a few cases in which they are predominant in the images (see for example Figure 8).

The drawback of our correction technique (in its current version) is a slight degradation of feature resolution, which manifests as blurring and dampening of LAC values of small metallic objects, and in some cases (especially in the first energy channel) as cupping artifacts in homogeneous materials. This could potentially be prevented by including more training data with objects of more diverse shapes and material compositions. Alternatively, the introduction of an objective function in the loss function minimization during the CNN training could identify and segment geometric features that are typically less artifact-prone in the intermediate energy channels and project them into the lower energy channel images.

Note that, as opposed to the simulated data, the high-energy channels are dominated by noise due to low photon statistics, indicating an eventual mismatch in the spectrum modeling. Interestingly, a circle shaped artifact (marked with a red arrow) that appears in the first energy channel image of Figure 8 is successfully removed by the MAR correction, possibly due to the absence of it in the higher energy channels.

The comparison of the 3D volumes of the input and corrected suitcases in Figure 9 highlights the overall result of the MAR correction. Note that each pair at a fixed energy channel is shown with identical value range of the color map, to emphasize the CNR comparison, as the overarching goal is the material discrimination and thus, contrast between the different objects enclosed in the suitcase. A significant improvement of the overall reconstruction quality is observed in the low-energy channel volume with only a moderate blurring drawback caused by the MAR correction (see Figure 9a). On the other hand, the correction does not seem to deliver a significant improvement in the intermediate energy channel (Figure 9b) as the noticeable streaking reduction from the CNN architecture does not reduce with the loss in feature resolution. We envisage that further developments in the architecture and eventual ad-hoc tuning of the training data to be similar to real suitcases can narrow the gap between our results and ideal corrections.

## 4. Conclusions

We have presented a method for metal artifact reduction in spectral X-ray CT reconstructions. As a part of our method, we introduced the concept of a spectral CNN architecture. Our approach learns features from the energy domain and thus extends the range of the problems that we can successfully correct for. The main advantage of our method is that it can be generalized and adapted for the problem at hand as the a priori information required for the correction is intrinsically incorporated into the simulated training data. The correction step for real measured data is then parameter-free and with near real-time speed, satisfying the requirements of real scanners. The performance of our method depends highly on the quality of the training data and how well its variety generalizes the problem. We found that training data generation can be a slow and in some cases complex procedure that needs to be tailored according to the desired application. However, this aspect becomes simpler in cases where the diversity of the objects under inspection decreases. In our examples mimicking use cases in luggage scanning for airport security, our correction always successfully improved the results. The current limitation of our correction method is the introduction of mild blur in the reconstruction, which is mainly due to computational hardware limitations. Due to the extremely large size of spectral reconstructions (3- or 4-D), the input data was downsampled in size losing high-resolution features. Likewise, the spectral CNN architecture was relatively limited in the layer depth and in the number and size of the convolutional filters (weight tensors). For these reasons, high-resolution scanners with a large data size may require further adaptations of the architecture or fragmentation of the datasets into smaller patches followed by a successive merging.

## Figures and Tables

**Table 1:** Performance of the CNN MAR correction measured as the RMSE, NRMSE and MSSIM, calculated between the input ground truth and CNN corrected data. The results are reported for the uncorrected data and corrected data training the CNN for 50 and 250 epochs.

Data	RMSE	NRMSE	MSSIM
Uncorrected	0.0035	0.12	0.926
50 epochs	0.0031	0.13	0.978
250 epochs	0.0020	0.09	0.990
